# Ten simple rules for providing bioinformatics support within a hospital

**DOI:** 10.1186/s13040-023-00326-0

**Published:** 2023-02-23

**Authors:** Davide Chicco, Giuseppe Jurman

**Affiliations:** 1 Institute of Health Policy Management and Evaluation, University of Toronto, 155 College Street, M5T 3M7 Toronto, Ontario Canada; 2 Data Science for Health Unit, Fondazione Bruno Kessler, Via Sommarive 18, 38123 Povo, Trento, Italy

**Keywords:** Supervised machine learning, Computational validation, Recommendations, Data mining, Best practices in machine learning

## Abstract

Bioinformatics has become a key aspect of the biomedical research programmes of many hospitals’ scientific centres, and the establishment of bioinformatics facilities within hospitals has become a common practice worldwide. Bioinformaticians working in these facilities provide computational biology support to medical doctors and principal investigators who are daily dealing with data of patients to analyze. These bioinformatics analysts, although pivotal, usually do not receive formal training for this job. We therefore propose these ten simple rules to guide these bioinformaticians in their work: ten pieces of advice on how to provide bioinformatics support to medical doctors in hospitals. We believe these simple rules can help bioinformatics facility analysts in producing better scientific results and work in a serene and fruitful environment.

## Introduction

Recent trends worldwide have shown that creating scientific research centres within hospitals can be advantageous both for the hospitals and for the scientific institutions [1, 2]. That is, medical doctors actively participating in biomedical research provide better treatments and therapies to patients than medical doctors who are uninvolved in scientific research; moreover, biomedical researchers working within a hospital produce better scientific results and outcomes than those working outside a hospital [3].

Bioinformatics and computational biology have become the pillars of scientific research programs carried out within hospitals, together with the old-fashioned, fundamental wet lab biological research [4]. Many hospitals worldwide have thus decided to create bioinformatics facilities where professional bioinformaticians, computational biologists, and biostatisticians can support medical doctors and physicians in their scientific projects [5, 6]. Biostatisticians usually provide hints and advise on how to perform a statistical analysis for a medical study, while bioinformaticians not only give advice, but also perform the computational biology analysis itself.

Once the bioinformatics facility is set up, its members typically get contacted by a medical doctor of the hospital asking them to perform a particular computational analysis on their patients’ dataset, by a specific deadline. The bioinformatician then analyzes these data through the bioinformatics tools and software they consider more adequate, and eventually delivers them to the medical doctor in a technical report document. Afterwards, if the results of these analyses look interesting to the medical doctor, they might decide to include them as co-authors in a scientific article to be submitted to a biomedical journal, or to recognize their support in the Acknowledgements section of the article.

In this study, we walk in the shoes of analysts hired in a bioinformatics facility of a hospital, and provide ten simple rules on how to perform their work, based on our experience and past mistakes. Few articles published recently already suggested some pieces of advice for providing bioinformatics support and to form a bioinformatics facility team [7, 8], but their contents are general, and not specific to hospitals. We propose these ten simple rules specific for hospitals: while they are far from being perfect or definitive, we believe our ten suggestions can help generate better scientific research and help bioinformaticians increase their awareness about their roles and work in a more serene work environment. Overall, these ten simple rules can also be viewed as a data science summary: a few good practices that, although consolidated and well rooted into commons sense, might still need to be recalled occasionally to improve the everyday activities of bioinformaticians in hospital environment.

## Rule 1: have a lot of patience

Working with medical doctors and other bioinformaticians is a great privilege, allowing the application of bioinformatics skills to real problems directly related to the health of real patients. No simulations here, no synthesized data, no theoretical stuff: actual lives. Although interesting and exciting, this activity can also be challenging. Medical doctors, in fact, might have unconventional time schedules and busy agendas. For example, they might set up a plenary meeting at 7:00am in the morning, requiring you to wake up and go to work several hours earlier that day. Another time they may need to cancel an important meeting with you due to a patient’s medical emergency.

In situations like these and for all the time you spend in the hospital, we recommend having a lot of patience.

Moreover, it is important to reaffirm that not only medical doctors might have a different mindset and a different agenda from yours, but also computational technicians, wet lab technicians, nurses, administrative staff probably see the world from a different perspective that you might not immediately grasp: patience and empathy are fundamental tools to use when interacting with them.

Have the capability to understand that a hospital is a work environment unlike any other in the world, and so your agenda and expectations need to be different from all the other places you worked in the past.

## Rule 2: always preserve the privacy of the patients and demand the same from all your collaborators, including medical doctors and hospital directors

Patients are the most important people in the hospitals: they are those for whom the hospitals were built. Although their interests are pivotal, they of course do not participate in the meetings between the bioinformatics facility analysts and the medical doctors. Therefore, it is crucial that during these meetings:

1. Everyone does their best to improve the conditions of patients.
2. Everyone discloses any potential conflict of interest beforehand.
3. Everyone respects patients’ privacy.

In modern hospitals, all employees must follow strict rules to preserve the privacy of patients. These rules include never disclosing patient information to strangers over the phone, and never placing patient data on Universal Serial Bus (USB) flash drives.

Laws call patients’ data *protected health information* (PHI), which includes: the individual’s past, present, or future physical or mental health or condition; the provision of health care to the individual; the past, present, or future payment for the provision of health care to the individual, and that identifies the individual or for which there is a reasonable basis to believe can be used to identify the individual [9]. Breaches of patient privacy, that means disclosure of private health information of patients outside the hospital, can cause any number of bad consequences to the patients themselves, including: loss of trust in the medical doctors, in the medical treatment, in the hospital, and in traditional medicine in general; humiliation; loss of employment; damage to reputation or relationships; problems with business or professional opportunities; negative effects on credit record, and more [10].

Unfortunately, despite all the patient privacy policies, confidentiality breaches still happen worldwide. A study by Beltran-Aroca and colleagues [11] analyzed data of patient confidentiality breaches in Spain and noted: “Most of the reported incidents were observed in public areas (37.9%), such as corridors, elevators, cafeteria, stairs, and locker rooms” [11]. Another older study by Mlinek and colleagues [12] stated that more than 53% of the analyzed breaches happened in the triage/waiting area of the emergency department of the hospital.

So, here is the rule we suggest for you to follow: always comply with all hospital privacy policies. Before working with patients’ data, ask to your department’s director which privacy policy or agreement is associated with them, and which authorization from the Research Ethics Board (REB) was granted. Investigate all the privacy rules, at each level: the hospital rules, the department rules, and the international rules in force (for example, the General Data Protection Regulation – GDPR in Europe).

Study these policies well, follow them strictly, and demand the same from everyone around you, including medical doctors and the hospital directors.

If you see a confidentiality breach, report it immediately to the privacy office. If necessary, contact a privacy lawyer to seek professional legal advice. Finally, we only mention here the relevance of warranting and/or improving the robustness of the bioinformatics platforms and infrastructures to protect against cyber-attacks [13]. Advanced information technology (IT) infrastructures protected by multiple layers of firewalls and multi-factor authentication modes can improve the safety of the patients’ data. Although, cyber-security is beyond the scope of this study, but we reaffirm its importance.

Techniques for de-identification of the PHI should be employed by the hospital, too [14]; if absent, you might propose their usage to your team leader or department director. In case of imbalanced datasets, bioinformaticians can consider generating and using synthetic data [15].

## Rule 3: keep in mind that the bioinformatics facility should support medical doctors, and not vice versa

Bioinformatics facilities are funded specifically to provide a supporting role to the research of the medical doctors. However, as time goes by and projects keep everyone busy, the funding principles of the bioinformatics facility might fall into oblivion, thus we believe it is important to reaffirm this fundamental idea: the bioinformatics facility was established and funded for sustaining medical doctors, and not vice versa. Physicians are not there to enhance your career, and to help you secure more publications, grants, or personnel: they work there to cure patients and to discover something scientifically meaningful to improve cures and therapies.

As in any scientific field, your career and your scientific goals are based on the scientific publications, for the most part. Depending on your institution’s policies, you might be included as coauthor in scientific articles to which you contribute, and most of these studies are built on ideas of the physicians for which you provided bioinformatics support.

Often there might be problems such as delays in the paper submissions, lack of timely communication, and changes of plans, and you may become frustrated because the delay in the submissions of articles might negatively affect your résumé and thus your career as well. In any case, always remember that your main job is to support medical doctors.

One way to keep being productive in terms of publications without depending on medical doctors – if the facility board allows it – is to dedicate some time to your own independent projects. In these studies, you and your collaborators can set your own pace, and go directly to the paper submission phase without having to wait for external approvals.

## Rule 4: always do what is best for patients

Patients are the most important people in a hospital, and your activity as a researcher or bioinformatician should always be focused on what is best for them. In principle, everybody agrees with this statement, but there are times when pursuing this task is difficult. Sometimes you might complete a complex analysis, be ready to submit your results to the medical doctors, and realize there is another bioinformatics method you did not use and that might lead to better results. Even if you were tired and thought your analysis results were sufficient, we would recommend always asking yourself: “What is best for patients?”.

If there is the chance that the new analysis might lead to more robust results, that would somehow impact the patients, of course we suggest you go for it. Whether small or big choices, “What is best for patients?” should be your guiding light in any decision-making process.

Be aware of individuals who give you suggestions that have nothing to do with scientific research but are politically motivated. For example, a few years ago someone we know was recommended not to use a specific software tool for their analysis because it was published by a *competitor* research group. The same happened to a former colleague of ours regarding a particular dataset: the principal investigator told him not to use a particular dataset, even if it was the state-of-art most recent one, because it was from an adversarial scientific institution.

Though these recommendations are of course unethical, they can be hard to reject, especially for junior researchers and subordinate employees out of positions of power. In such cases, however, we again recommend you consider this important question (“What is best for patients?”) and then behave accordingly.

Even if you noticed that something wrong is happening, you might not know what to do without risking your jobs or reputation. In cases where you see something unethical or illegal, our suggestion is to discuss it with your team leader or principal investigator as early as possible. If they decided to let the issue go, it could be advisable to contact a lawyer outside your work organization and get their honest feedback about the event. In any case, the worst thing to do is to pretend that nothing happened.

## Rule 5: before starting to work on a scientific project, clearly define its scientific goals with the medical doctors

Your role, working in the computational biology facility, is to perform bioinformatics analyses on datasets which are given to you by the medical doctors and their teams. The dataset therefore is the key element, but alone is insufficient. To carry on a complete scientific study, another ingredient must be present: the scientific goals of the study. The goals of the study should be stated clearly, and should be detailed and precise. So, when you are asked by physicians to analyze data, invite them to sit down with you and your team to discuss the desired scientific goals. What do we expect to see at the end of the analysis? What is the scientific hypothesis? What does the scientific literature say about these goals? Were similar analyses published in other scientific studies recently? Are we foreseeing an advancement with respect to the state of the art on that medical field? If the scientific goals look too generic and broad to you, ask the medical doctors to provide more details and to come up with a precise, detailed, thorough scientific question.

Some years ago a former colleague told us that she was contacted by a medical group in the hospital where she used to work. They asked her to analyze data of electronic health records through traditional biostatistics techniques “to see what comes out from there”. She refused to do anything until they provided more precise and detailed information about their scientific goals and explained which statistical trends they expected to see in the data. She made the right decision: a poorly-defined scientific question can produce a waste of time, energy, enthusiasm, money, and human resources. On the contrary, a well-designed scientific question is the foundation of a scientific study.

Moreover, a bioinformatician should feel free to share their point of view regarding scientific goal definition and experiment design, allowing the project to be arranged in a common frame. The experience in past bioinformatics studies can provide interesting feedback to medical doctors on how to carry out the current project with the bioinformatics facility: there should not be a single direction of communication (that is, medical doctors telling bioinformaticians what to do), but rather an exchange of ideas between the bioinformatics team and them.

The bioinformatics facility members should also initially enquire about the sense of the data to analyzed, asking how these data were collected, with which technology, so that they can discuss the feasibility of the project with the medical doctors. Physicians sometimes might have a partial or incomplete understanding about what can be done or undone with some specific dataset; it is important that this aspect is clarified at the beginning.

Information about the desired format of the output should be decided before starting a project, too (Rule #7). Ask the medical doctors what format they would like the analysis output delivered in (plot images, tables of rankings, etc.), and maintain the scientific interaction with the clinicians throughout the whole study to ensure the outcomes are fairly grounded.

## Rule 6: keep in mind that the simplest computational methods might be the best solution for a scientific problem

When facing a particular scientific problem, computer scientists sometimes tend to choose complicated and unusual computational methods rather than picking simple techniques. We do not know why; it is possible that it might have something to do with originality: some computer scientists might think that the more creative the solution is, the more interesting the results can be. This is even more common in the dissemination phase: sometimes unnecessary complex descriptions are written to explain quite simple algorithms, as if the usage of vacuously intricate jargon might add value to the methods (spoiler: it does not).

Instead, we recommend doing the opposite: when deciding which methods or tools to use, always start with the simplest ones. In the worst case, they provide a performance base line for future comparisons. Similar to what is suggested for machine learning [16], a simple method can be a good starting solution for a bioinformatics analysis because it allows the bioinformatician to keep everything under control and to understand each step of it.

For a bioinformatics study which involves supervised machine learning, for example, we suggest starting with linear regression [17], For bioinformatics projects which include unsupervised machine learning or clustering, we recommend beginning with *k*-means [18]. For survival analysis, the traditional Cox regression [19] could be a good starting point. For dimensionality reduction, the principal component analysis (PCA) could be a good first step before moving to more advanced visualization techniques such as t-distributed stochastic neighbor embedding (t-SNE) [20] and uniform manifold approximation and projection (UMAP) [21].

If the results generated by the simplest methods are insufficient, apply more sophisticated techniques, of course. But do not start with complicated stuff: start with the simple stuff.

Moreover, the usage of simple methods would facilitate the explainability of the results: results obtained with easier computational techniques would be more likely to be understood and interpreted by clinicians, including ones with a limited informatics education.

Before exploring machine learning or computational statistics methods to make new discoveries in the dataset, we recommend to perform an exploratory data analasys (EDA) [22, 23] to get a sense of the data analyzed.

## Rule 7: if possible, deliver your results in the form of tables, figures, and text ready for inclusion in an article draft

Once you clarified the scientific goals of the study (Rule #5), and you conducted the bioinformatics analysis requested by the medical doctors, you have to decide how to deliver your results. A typical way to deliver bioinformatics results within hospitals is to write down a technical report, usually by mixing technical writing, scientific writing, and informal writing. This document can be prepared with LibreOffice Writer and delivered as a PDF file.

One might tend to include tables and figures as they are generated from the bioinformatics software programs, but we suggest an extra step: prepare these visual elements in a way that they are ready for inclusion in the scientific article draft. If the tables and figures turn out to be the final ones to be included in the manuscript, one would not need to repeat the analysis again. Figures ready to be included in a paper draft would have the proper labels for each visual element, written with a font size large enough to be read by anyone, and positioned in a non-messy way within the image [24]. The captions of each figure would contain all the possible information to make figure self-complete and independent from the text: a reader might decide to just see that figure, read only its caption and should be able to get a complete piece of information about the study [24]. Tables should be made complete, too, with all the columns explained in the tables’ captions [25].

Preparation of tables and figures is by no mean an empirical task: nowadays data visualization is a firmly grounded subject, endowed by a robust theoretical background driving the construction of an effectively impacting figure and table. However, a number of shared rules of thumb do exist to help bioinformaticians in preparing good charts for scientific purposes, and they do not require a deep knowledge of the cognitive aspects of visualizations to be put into practice [24].

Repeating the same bioinformatics after months or even years can be tricky: one might have forgotten some minor steps or, more importantly, software programs used may have changed or been updated to new versions, which generate slightly different results from the results obtained previously.

Moreover, if tables and figures are ready for inclusion in the article, its authors will be able to proceed to the submission earlier, without additional delays should they have to wait for new versions of tables and figures.

Even if figures and tables can be considered final, you should arrange your computational work environment (software scripts, notebooks, etc) so that the bioinformatics analyses can be repeated easily at any time. In fact, it is likely that you will need to re-execute your scripts several times, possibly with different subsets, or with the same dataset if a previous mistake was noticed. To this end, it is important to keep your software programs and scripts up-to-date, documented, and saved through version-control tools such as Git or similar [26–31].

## Rule 8: look for an alternative validation dataset of the same type and disease to repeat your analysis

The Open Data and FAIR principles have found large usage in bionformatics [32–34], with Gene Expression Omnibus (GEO) [35, 36], ArrayExpress [37–39], The Cancer Genome Archive (TCGA) [40], and Sequence Read Archive (SRA) [41–43] being central, popular resources for open data of gene expression. Biomedical datasets can also be found on the University of California Irvine Machine Learning Repository [44], on Kaggle [45], on Google Dataset Search [46], and on Re3data [47].

This huge availability of datasets allows bioinformaticians worldwide to perform secondary analyses to verify their main findings on validation cohorts. We suggest you take advantage of this possibility and to try to repeat your bioinformatics analysis on one of these public bioinformatics datasets, if possible. Of course, you need to look for datasets of the same disease (for example, neuroblastoma [48]), cohort status (for example, diagnostic or prognostic), and of the same data type (for example, single-cell RNA-seq [49]). Keep in mind that datasets of the same disease might be found by using different keywords: for example, the terms *Phyllodes tumor*, *ductal carcinoma in situ*, and *angiosarcoma* all refer to *breast cancer*, but might not be found if you just look for the “breast cancer” keyword.

There are several tools to retrieve datasets from these resources [50–53]. Finding an alternative validation cohort where you can repeat your bioinformatics analysis can be a pivotal step to obtain further confirmation about the findings you made on the original primary cohort’s data. Additionally, it will make the final study article more robust.

Caveat emptor: even if all the aforementioned constraints are satisfied, the validation cohort may still fail in confirming the outcome obtained in training because of distribution shift, that is the different shape in distribution between datasets, affecting many studies worldwide.

## Rule 9: keep in mind that your lack of education on medicine might actually make your analysis results more unbiased and objective

If one works in a bioinformatics facility, they probably have an academic degree in computer science, computational biology, or a similar field. It is very likely that the bioinfomatician never studied much about medicine in their life, and their medical knowledge might then be limited. But what can look like a flaw and a limitation, might actually turn out to be an asset: if you performed your bioinformatics analysis by treating all the clinical features in the same unbiased way (the so-called *data driven* approach), you would avoid adopting the potential biases that the medical doctors picked up after several years of practice. Cognitive biases, in fact, can influence medical decision making [54–56] and sometimes lead to poor results [57]. On the other hand, lack of medical education, can lead to more unbiased results, that will eventually lead to better medical decision making. Bioinformatics is based on computer science, and computer science integrated with biology can help produce more “evidence-based medicine” and less “eminence-based medicine” [58].

If a bioinformatician has an opportunity to study medicine, of course we recommend they take it. However, what we are saying here, however, is that a computer-based, medical knowledge-agnostic, bioinformatics approach can be an asset for a medical study, because it can generate more unbiased results.

Even if the lack of medical education should not scare computational biologists, we reaffirm the importance of a continuous, frequent interaction and exchange of ideas between them and medical doctors, for all the duration of the project. Regular video-meetings, for example every two weeks, can help avoid nonsense tested hypotheses or correct wrongly imposed assumptions, saving time both to bioinformaticians and physicians, and helping generate more robust results.

Additionally, we invite bioinformaticians to have an initial open, honest discussion with the medical doctors involved in the project to talk about any possible biases they might have: identifying the potential sources of bias in a project design beforehand would turn to be very useful later on when the bioinformaticians and the medical doctors have to comment the results [54, 55, 59]. This way, you and the authors of the study will be able to understand how general your scientific discoveries are, or if they are tied only to a particular subpopulation of patients.

## Rule 10: use and encourage usage of open source software and publication in open access journals

As a member of the bioinformatics facility, you should have almost full control of the software programs and of the programming languages you use for your analyses. If so, we suggest you to always use open source programming languages (such as R and Python), open source software package platforms (such as Bioconductor [60] or Bioconda [61]), and open source software programs (such as Galaxy [62]), while avoiding proprietary software [16]. Using open source software would be beneficial for you in multiple ways, such as the possibility to freely share your code pieces and results with your collaborators and to publish them online on GitHub or GitLab, without worrying about the programming language license constraints.

While you might be able to decide which software to use, you probably will not be in the position to decide to which scientific journal to submit the bioinformatics study article. However, even without the last word, we suggest you recommend that the authors of the study choose an open access journal. The publication of an article on an open access journal has many benefits, including the possibility to make an article accessible to anyone worldwide, including people in the least developed countries. Moreover, open access publications can have more impact on scientific literature, and therefore usually collect more citations. A list of relevant bioinformatics open access journals can be found on the Scimago Journal Rankings website [63, 64].

## Conclusion

Since the early 2000s, bioinformatics has become crucial for biomedical research: most physicians have understood the importance of analyzing genomics and proteomics data of patients computationally to identify trends and correlations. Bioinformatics findings can be integrated with discoveries made through traditional laboratory test results and biomedical engineering machines exams (such as magnetic resonance imaging and computed tomography scan, for example) and help medical doctors have a clearer understanding about the prognosis of the patient, resulting in better medical decision-making.

As a result, hospitals worldwide have started to create and fund bioinformatics facilities, where computational biologists can be contacted by physicians to run specific analyses on patient datasets. While the role of these analysts can be pivotal, almost nobody receives formal training for these recently-established roles. We therefore designed and wrote these ten simple rules to give some guidance to analysts working in these facilities, and allow them to avoid common mistakes that we might have made in the past. So far, we have worked on a fair number of scientific projects, and we agree that *working smart* is definitely more important than *working hard*.

Caterina Fake once wrote: “So often people are working hard at the wrong thing. Working on the right thing is probably more important than working hard” [65]. We agree with this quote and would add: working on the right things and in the right ways is probably more important than working hard.

## Data Availability

Not applicable.

## Abbreviations

EDA: Exploratory data analysis
EHRs: Electronic health records
GDPR: General Data Protection Regulation
GEO: Gene Expression Omnibus
MD: Medical doctor
PCA: Principal component analysis
PDF: Portable Document Format
REB: Research Ethids Board
SRA: Sequence Read Archive
t-SNE: t-distributed stochastic neighbor embedding
TCGA: The Cancer Genome Archive
UMAP: Uniform manifold approximation and projection
